# A novel bispecific antibody targeting CD3 and prolactin receptor (PRLR) against PRLR-expression breast cancer

**DOI:** 10.1186/s13046-020-01564-4

**Published:** 2020-05-12

**Authors:** Yuexian Zhou, Huifang Zong, Lei Han, Yueqing Xie, Hua Jiang, John Gilly, Baohong Zhang, Huili Lu, Jie Chen, Rui Sun, Zhidi Pan, Jianwei Zhu

**Affiliations:** 1 Engineering Research Center of Cell & Therapeutic Antibody, MOE,Shanghai Jiao Tong University, Dongchuan Road, Shanghai, China; 2 School of Pharmacy, Shanghai Jiao Tong University, Dongchuan Road, Shanghai, China; 3 Jecho Laboratories, Inc, Frederick, MD, USA; 4 Jecho Biopharmaceuticals Co., Ltd, Tianjin, China

**Keywords:** Bispecific antibody, CD3, PRLR, Breast cancer

## Abstract

**Background:**

Prolactin receptor (PRLR) is highly expressed in a subset of human breast cancer and prostate cancer, which makes it a potential target for cancer treatment. In clinical trials, the blockade of PRLR was shown to be safe but with poor efficacy. It is therefore urgent to develop new therapies against PRLR target. Bispecific antibodies (BsAbs) could guide immune cells toward tumor cells, and produced remarkable effects in some cancers.

**Methods:**

In this study, a bispecific antibody targeting both tumor antigen PRLR and T cell surface CD3 antigen (PRLR-DbsAb) was constructed by split intein mediated protein transsplicing (BAPTS) system for the first time. Its binding activity was determined by Biacore and Flow cytometry, and target-dependent T cell mediated cytotoxicity was detected using LDH release assay. ELISA was utilized to study the secretion of cytokines by immune cells. Subcutaneous tumor mouse models were used to analyze the in vivo anti-tumor effects of PRLR-DbsAb.

**Results:**

PRLR-DbsAb in vitro could recruit and activate T cells to promote the release of Th1 cytokines IFN- *γ* and TNF- *α*, which could kill PRLR expressed breast cancer cells. In xenograft models with breast cancer cell line T47D, NOD/SCID mice intraperitoneally injected with PRLR-DbsAb exhibited significant inhibition of tumor growth and a longer survival compared to mice treated with PRLR monoclonal antibody (PRLR mAb).

**Conclusions:**

Both in vitro and in vivo experiments showed PRLR-DbsAb had a potential therapy of cancer treatment potential therapy for cancer. Immunotherapy may be a promising treatment against the tumor target of PRLR.

## Background

PRLR is one kind of type I cytokine receptors highly expressed in breast cancer cells [1]. It is only slightly expressed in normal breast tissues while highly expressed in tumor breast tissues [1–3]. Engineered monoclonal antibodies (mAbs) are important therapeutic proteins [4]. Jason *et al* has reported that humanized anti-PRLR antibody could inhibit the dimerization of PRL and its receptor PRLR, which subsequently could inhibit the tumor cell proliferation that mediated by its downstream signaling effectively [5]. The blocking PRLR antibody has shown a very good safety profile in phase I clinical trials [6]. In addition, an anti-PRLR antibody-drug conjugate (ADC) had significant PRLR-specific antitumor activity against breast cancer [7], and bispecific antibody-ADCs bridging HER2 and PRLR improved efficacy of HER2 ADCs [8]. Therefore, PRLR is considered to be a tumor associated antigen (TAA) with a high potential in clinical applications. However, the PRLR antibody is showed to be lack of efficacy in clinical trials despite of its favorable pre-clinical data [9]. Tumor immunotherapies including immune checkpoints [10,11], CAR-T [12], oncolytic virus [13] and bispecific antibodies [14] are proved to be effective anti-tumor treatments. The PD-1/PD-L1 checkpoint blockade has significant progress in melanoma, lung cancer, and lymphoma [15,16], and a number of clinical trials in breast cancer and glioma are also being efficiently carried out worldwide [17,18]. Bispecific antibodies targeting the CD3 antigen, which could recruit T cells to tumor cells to enhance cytotoxicity, are demonstrated to have both good pre-clinical and clinical potency. Currently there are two CD3-bispecific antibodies approved for treatment, one is BiTE-based CD3/CD19 (Blinatumomab) [19] for the treatment of B cell acute lymphoblastic leukemia and the other is Triomab-based CD3/EpCAM (Catumaxomab) [20] indicated for malignant ascites caused by EpCAzM+ cancer cells. Moreover there are many other clinical trials with bispecific antibodies for the treatment of solid tumors and hematological tumors based on other tumor antigens such as CEA [21], HER2 [22], EGFRvIII [23], EGFR [24] and CD20 [25]. It is reported more than 60 structures have been developed for the bispecific antibodies, including symmetric and asymmetric structures based on IgG fragments and types used [26]. Recently our lab has developed a novel universal platform for generating IgG type bispecific antibodies (BAPTS). The platform is based on split intein, which could solve the mismatch between light and heavy chains with high efficiency through its trans-splicing function. The CD3/HER2 bispecific antibody generated with this method showed a good affinity for its targets and a favorable pharmacokinetic profile, as well as a significant anti-tumor activity [27]. In this research we generated a bispecific antibody PRLR-DbsAb targeting both PRLR and T cell surface antigen CD3 by BAPTS platform. In vitro PRLR-DbsAb efficiently inhibited the growth of breast cancer cells with high PRLR expression, accompanied with T cell activation and cytokines release. In vivo it promoted the infiltration of immune cells that subsequently inhibited the tumor development and extended the survival time of mice. As a result, PRLR-DbsAb could be a new treatment for breast cancer.

## Materials and methods

### Mice and tumor cell lines

Female NOD/SCID mice were purchased from Charles River Laboratories in China and handled according to guidelines from the Institutional Animal Care and Use Committee of the School of Pharmacy of Shanghai Jiao Tong University. MDA-MB-231, MCF-7 and SKBR-3 cells were obtained from American Type Culture Collection, and T47D and Jurkat cells were purchased from Chinese Type Culture Collection. All cell lines were cultured under standard conditions and used within 6 months after resuscitation without re-authentication.

### Expression and purification

The PRLR-DbsAb was generated by BAPTS system, which was previously described in detail [27*,*^28^]. CD3 antibody-fusion protein (Fragment A) was expressed in a stably transfected cell line CHO while PRLR antibody-fusion protein (Fragment B) was expressed in 293E cells using transient gene expression (TGE) technology. Both Fragment A and Fragment B were purified by Protein L affinity chromatography (GE Healthcare). The monoclonal antibodies used in this study including PRLR antibody (PRLR mAb), CD3 antibody (CD3 mAb) and PD-1 antibody (PD-1 mAb) were expressed by 293E cells and purified by Protein A affinity chromatography (GE Healthcare). The affinity of CD3 mAb and PD-1 mAb to their antigens was previously confirmed [27*,*^29^]. The amino acid sequence used for the PRLR mAb is identical to that of the PRLR monoclonal antibody LFA102 [5]. All recombinant antibodies were dialyzed overnight to phosphate buffer saline (PBS) and sterilized by filtration using a 0.22 *μ*m filter.

### Flow cytometry

#### a Evaluating the relative levels of PRLR and PDL1 expressed on cell membrane

Breast cancer cell lines (MDA-MB-231, MCF-7, SKBR-3, and T47D) were incubated with control isotype IgG, PE-conjugated mouse anti-Human PRLR (Sino Biological) or APC-conjugated mouse anti-Human PD-L1 (Sino Biological) on ice for 30 min and then washed twice with FACS buffer (PBS with 2% FBS). Samples were analyzed with CytoFLEX cytometer. In this experiment that combination of PRLR-DbsAb and PD-1 mAb was used to investigate the killing effect of effector cells on target cells, the supernatant of the culture medium was removed after centrifugation at 300 x g for 5 min and the bottom cells (including effector cells and target cells) were harvested by digesting with 0.25% trypsin. The harvested cells were incubated with APC-anti human PD-L1 antibody on ice for 30 min.

#### b Binding of PRLR-DbsAb to T lymphocyte

Human peripheral blood mononuclear cells (PBMCs) isolated from a healthy volunteer were simultaneously incubated with three antibodies of 5 *μ*g/ml PRLR-DbsAb, anti-human CD4 (BD Biosciences) and anti-human CD8 (BD Biosciences) on ice for 30 min. Samples were washed twice with FACS buffer and analyzed with CytoFLEX cytometer using CytoExpert software.

#### c Binding of PRLR-DbsAb to T47D cell

T47D cells were resuspended in FACS buffer on ice and incubated with PRLR mAb in different concentration or PRLR-DbsAb with 5 *μ*g/ml, followed by PE-conjugated goat anti-Human IgG-Fc antibody (eBioscience^TM^). Samples were analyzed with CytoFLEX cytometer using CytoExpert software.

### Affinity measurement of PRLR-DbsAb

The affinity of PRLR-DbsAb, PRLR mAb and CD3 mAb was determined using surface plasmon resonance (Biacore T200, GE). The extracellular domain of human PRLR (Sino Biological) and CD3D/CD3E heterodimer (Sino Biological) were immobilized to a CM5 chip surface using standard 1-ethyl-3 (3-dimethylaminopropyl) carbodiimide (EDC)/N-hydroxysuccinimide (NHS) amine coupling protocols. The concentration series were fit to a 1:1 binding model to determine the binding (Ka) and dissociation (Kd) rate constants and the equilibrium dissociation constant (KD). To demonstrate simultaneous binding, the PRLR extracellular domain was coupled to a CM5 sensor chip as described above. The PRLR-DbsAb was injected for 2 min followed by a 2 min injection of CD3D/CD3E heterodimer. Surfaces were regenerated using injections of 0.1 M glycine, pH 1.5. PRLR mAb was injected as a control.

### Analysis of T-cell redirection

CD3-positive Jurkat cells were labeled with carboxyfluorescein succinimidyl amino ester (Invitrogen) according to the manufacturer’s instruction. The labeled Jurkat cells and PRLR positive T47D cells were mixed at equal ratio then treated with PRLR-DbsAb or PRLR mAb (100 ng/ml) for 30 minutes at 4 ^∘^C. Light microscopy was performed to detect T cell redirection.

### Analysis of T-cell activation

Early signs of T-cell activation (CD69) were detected by FACS after bispecific antibody treatment was initiated. Freshly thawed PBMCs were treated with PRLR-DbsAb or PRLR mAb (100 ng/ml) and placed in 96-well plates with or without target cells at an effector-to-target (E/T) ratio of 10:1. The culture supernatant was collected to detect secreted IFN- *γ*, TNF- *α* and IL10 level by Immunoassay Kit (Multi Sciences). Cells were also harvested and analyzed for activation with anti-human CD4, anti-human CD8 and anti-human CD69 antibodies (BD Biosciences).

### In Vitro cytotoxicity

In brief, target cells were seeded on 96-well cell culture plates and cultured overnight. Then antibodies at varying concentrations were pre-incubated for 30 minutes at 37 ^∘^C in culture medium (no phenol RPMI 1640+10% FBS) before adding the human PBMCs at a 10:1 E/T ratio or various E/T ratios (ranging from 2.5:1 to 20:1). After incubation for additional 20 hours, the supernatant was collected and the lactate dehydrogenase activity was measured using Cytotoxic 96 ⓇNon-Radioactive Cytotoxicity Assay Kit (Promega). The percentage of cytotoxicity was calculated as following: %cytotoxicity = (experimental lysis-spontaneous effector lysis-spontaneous target lysis)/(maximum target lysis -spontaneous target lysis) * 100.

### Xenograft studies

In vivo experiments were performed with 6 to 8-week-old female NOD/SCID Mice and all mice were first implanted in the subscapular region with 60-day release, 17- *β*-estradiol (0.72 mg) pellets (Innovative Research of America) before injecting tumor cells [30*,*^31^].

#### a Subcutaneous tumor plus subcutaneous PBMCs (E/T 1:4 mixing)

NOD/SCID mice embedded with 17- *β*-estradiol pellets were subcutaneously injected with a mixture of T47D cells (1* 10^7^) and PBMCs (2.5* 10^6^) isolated from the same donor in Hanks’ buffered saline solution at an E/T ratio of 1:4. On the second day, the recombinant proteins were intraperitoneally injected according to the schedule shown in Fig. 5a. Tumor size was measured twice a week with a vernier caliper and tumor volume was calculated using the formula approximated formula V = (length * width *width)/2. After the mice were sacrificed, the tumor weight was detected by the electronic analytical balance and the photographs of stripped tumors were taken.

#### b Subcutaneous tumor plus intraperitoneal PBMCs

T47D cells (1* 10^7^) cells in 0.1 ml Hanks’ buffer were subcutaneously implanted into mice. After 12 days, the tumor grew and the mice were randomly divided into two groups (*n*=3 in each group). The isolated PBMCs were intraperitoneally injected into mice, and next day 3 mg/kg PRLR-DbsAb and control solvent were administered intraperitoneally. Other procedures were similar to those described in the previous paragraph (a).

### IHC analysis of CD8 and PDL1

Tumor samples were fixed in 4% paraformaldehyde and embedded in paraffin. Sections of 3 *μ* in thickness were cut perpendicularly to the long axis of the tumor tissue for immunohistochemistry, and paraffin-embedded tissue sections were subjected to heat-induced antigen retrieval in 0.1 M Tris ·HCl buffer (pH 9.0) at 98 ^∘^C for 15 min. Endogenous peroxidase was blocked for 20 min with 3% H2O2 in PBS. Subsequently, the sections were blocked with normal goat serum, followed by incubating with antibodies against CD8 and PDL1 overnight at 4 ^∘^C. The sections were stained using a polymer HRP detection system (DAKO) and counterstained with hematoxylin. The sections were examined using an NIKON ECLIPSE C1 microscope.

### Immunofluorescence microscopy of CD8

The above sections were blocked with 5% BSA for 1 h at room temperature, then incubated with anti-human CD8 for 2 h at room temperature, followed by incubation with a secondary antibody conjugated with DyLight 488 (Earthox, Millbrae) at 37 ^∘^C for 1 h. Subsequently, the tissues were stained with DAPI to detect the cell nuclei. The coverslips were mounted onto glass slides, and the images were viewed with an XSP-C204.

### Statistical analysis

Differences between samples indicated in the figures were tested for statistical significance by the Student t test and *P* <0.05 was considered statistically significant.

## Results

### Generation and purification of PRLR-CD3 bispecific antibody (PRLR-DbsAb) using BAPTS platform

**Fig. 1:**
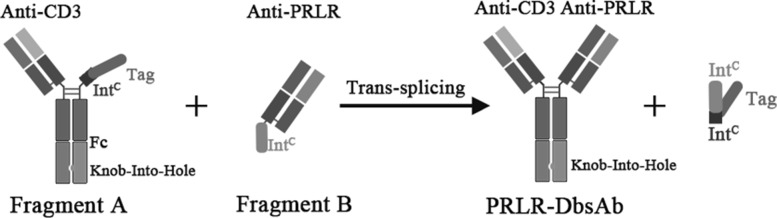
Schematic diagram of PRLR-DbsAb generated by BAPTS platform

To construct PRLR-specific bispecific antibody, we selected antibody variable regions from PRLR humanized antibody with proven preclinical activity and clinical safety. PRLR-DbsAb was generated by intein mediated trans-splicing of fragments A (anti-CD3) and B (anti-PRLR) (Fig. 1). Both antibody fragments were purified by Protein L affinity chromatography. Fragment A could be reduced into three peptides CD3Lc, CD3Hc and IntCFcH (Figure S1 b and c). Likewise, fragment B could be reduced into two peptides, PRLRIntN and PRLRLc (Figure S1a). After fragments A and B were cleaved and fused by intein Npu, a new band was observed on SDS-PAGE at positions corresponding to the expected size of the splice product PRLR-DbsAb (Figure S1b). PRLR-DbsAb was purified by Protein A affinity chromatography which removes fragment B from the reaction mixture (Figure S1d). PRLR mAb was purified by Protein A affinity chromatography (Figure S1e).

### PRLR-DbsAb cytotoxicity correlates with the target cell PRLR expression level

**Fig. 2:**
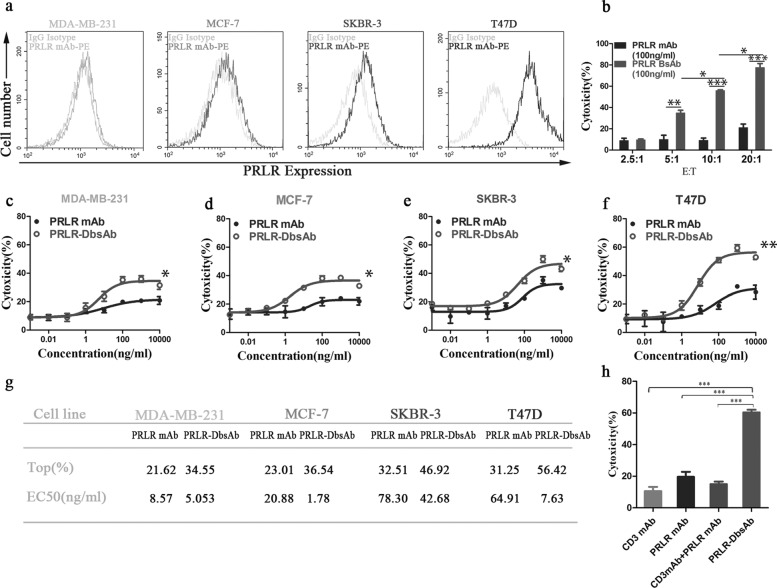
PRLR-DbsAb cytotoxicity correlates with the target cell PRLR expression. (**a**) FACS histograms of PRLR expression in different breast cancer cell lines (MDA-MB-231, MCF-7, SKBR-3, and T47D). Gray histogram, nonspecific IgG antibody fluorescence signal. (**b**) Cytotoxicity mediated by PRLR-DbsAb and control PRLR mAb with the same dose of 100 ng/ml at different ratios of effect cells to target cells (2.5:1-20:1). (**c**–**f**) Detection of LDH release after freshly isolated PBMCs were incubated with target cells at 10:1 (E: T) for 20 hours under different treatments. PRLR-DbsAb mediates T-cell killing of PRLR-expressing T47D cells with dose-dependent (**f**), and weaker killing of PRLR cells MDA-MB-231 (**c**), MCF-7 (**d**), and SKBR-3 (**e**). (**g**) The EC50 values and Top cytotoxicity were calculated by fitting the dose-response curve with Graphpad Prism software. (**h**) The combination of PRLR mAb and CD3 mAb did not mediate T cell killing of T47D cells. Three independent experiments were performed and the data were presented as the Mean ±SEM. *P <0.05, **P <0.01 and ***P <0.001

There is no study on the expression of PRLR in breast cancer tissues in china. To examine the extent of PRLR expression in breast cancers, immunohistochemistry was performed on commercial tissue chip. It was shown that the positive expression of PRLR in breast cancer tissues was significantly higher than that in adjacent tissues similar to normal breast tissues (Figure S2, Table 1). We also observed an increase in PRLR-positive breast cancer samples as tumor grades increased including pathological grade, clinical or N stage (Table 3). These results further provided a strong support for PRLR as drug target for breast cancer. PRLR is reported expressing in both HER2 negative (MDA-MBA-231 and MCF-7) and HER2 positive (SKBR-3 and T47D) cells [32]. The PRLR expression level in the four different cell lines mentioned above was assessed with flow cytometry and it was shown that they had various expression level with T47D having the highest followed by SKBR-3, MCF-7 and MDA-MB-231 (Fig. 2a). The cytotoxicity of immune effector cells mediated by PRLR-DbsAb depends on the ratio of effector cells to target cells, and PRLR-DbsAb demonstrated a significant T cell cytotoxicity as compared to PRLR mAb starting at low ratio of 5:1 (*P* <0.05) (Fig. 2b). The PRLR-DbsAb (100 ng/ml)-mediated killing activity of PRLR-high expression T47D reached 60% at a ratio of 10:1 (Fig. 2b). However, combination of CD3 mAb and PRLR mAb show lower cytotoxicity mediated by PBMCs than PRLR-DbsAb (Fig. 2h). It demonstrated that PRLR-DbsAb causes cytotoxicity through the synergistic effect of recruiting immune cells not the combined effect of CD3 mAb and PRLR mAb. PRLR-DbsAb increases cytotoxicity in all breast cancer cells with different PRLR-expression compared to control PRLR mAb group (Fig. 2, c-f). The best killing activities of PRLR-DbsAb to different breast cancer cell lines T47D, SKBR-3, MCF-7 and MDA-MB-231 were 56.42%, 46.92%, 36.54% and 34.55% respectively with the EC50 all being ng/ml scale (Fig. 2, a and g). All the results above indicated that T cell mediated cytotoxicity was correlated with the PRLR expression level.

**Table 1:** Patient characteristics of this study and PRLR expression

variable	Breast cancer tissue				Para-carcinama tissue			
	Total	-(*%*)	+(*%*)	++(*%*)	Total	-(*%*)	+(*%*)	++(*%*)
Female	30				28*			
Age(years)								
<55	23	2	14	7	23	20(87.0)	3(13.0)	0
>55	7	1	3	3	5	3(60.0)	2(40.0)	0
Pathology grade								
I+II	12	1(8.3)	8(66.7)	3(25.0)	15	14(93.3)	1(6.7)	0
II-III+III	18	2(11.1)	9(50.0)	7(38.9)	13	9(69.2)	4(31.8)	0
Clinical stage								
1+2	23	2(8.7)	15(65.2)	6(26.1)	21	17(81.0)	4(19.9)	0
3	7	1(14.3)	2(28.6)	4(57.1)	7	6(85.7)	1(14.3)	0
N stage								
0+1	24	3(12.5)	15(62.5)	6(25)	22	18(81.8)	4(18.2)	0
2+3	6	0	2(33.3)	4(66.7)	6	5(83.3)	1(16.7)	0

^*^Data missing due to two tissue fell out

-(*%*),+(*%*) and ++(*%*) represent negative, weak and positive expression of PRLR respectively

### Purified PRLR-DbsAb recruits T cells to PRLR-expression T47D cells

**Fig. 3:**
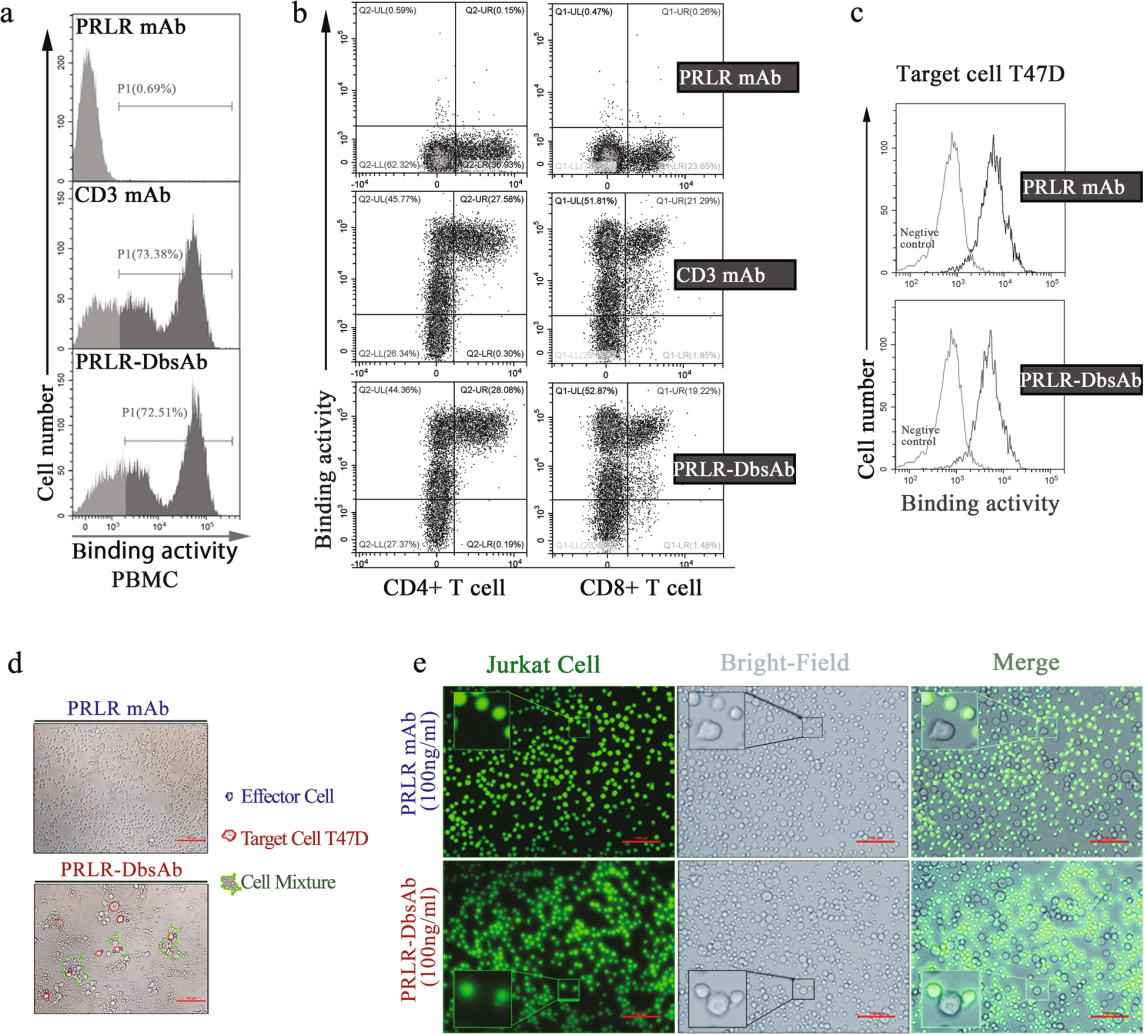
Purified PRLR-DbsAb redirects T cell to PRLR-expression T47D cells. PBMCs were incubated with 5 *μ*g/ml PRLR-mAb, CD3 mAb or PRLR-DbsAb for 30 min and then the APC-labeled anti-human Fc secondary antibody were simultaneously added with FITC-labeled anti-CD8 and PE-labeled anti-CD4 antibodies (**a** and **b**). (**a**) FACS analysis of PRLR-DbsAb binding to isolated PBMCs. The grey region represents the negative fluorescent signal; the black region is the positive fluorescent signal. (**b**) Flow cytometry analysis of PRLR-DbsAb binding to CD4 and CD8 positive T lymphocytes. (**c**) Flow analysis histogram of PRLR-DbsAb binding PRLR-positive cell line T47D. The PE-labeled anti-human-Fc antibody was added after incubating with PRLR mAb and PRLR-DBsAb, followed by FACS fluorescence detection. It was observed respectively with a light microscope and a fluorescence microscope that the PRLR-DbsAb redirected T cells (**d**) and T cell-derived CD3-positive tumor Jurkat cells (**e**) to T47D cells. The experiment was repeated three times

Next we investigated the mechanism of killing of breast cancer cells mediated by the bispecific antibody PRLR-DbsAb targeting CD3 and PRLR. The bispecificity of PRLR-DbsAb against cells expressing appropriate targets was analyzed with flow cytometry. PRLR-DbsAb had a high affinity for CD4+ and CD8+ T cells (both known to express CD3) from human PBMCs (Fig. 3, a and b) and specifically bound to PRLR highly expressed T47D cells (Fig. 3c). In addition, binding affinities of PRLR-DbsAb to recombinant PRLR extracellular domain (ECD) and CD3e were measured by surface plasmon resonance to be at equilibrium dissociation constants (KD) of 2.31E-9 and 8.36E-8 M respectively (Table 2). Importantly, PRLR-DbsAb (10 ng/ml) could recruit effector cells to T47D cells significantly compared with PRLR-mAb (10 ng/ml) according to the results obtained with microscopy (Fig. 3d). The CD3-positive Jurkat cells were also found to assemble around PRLR-expression T47D cells when PRLR-DbsAb was present at 10 ng/ml (Fig. 3e). The above results showed purified PRLR-DbsAb recruits T cells to PRLR-expression T47D cells.

**Table 2:** Relationship between PRLR expression and sample

Variables	Total	PRLR protein expression		*p*-value
	58	- (*%*)	+/++ (*%*)	
Breast cancer tissue	30	3(10.0)	27(90.0)	<0.05
Para-carcinoma tissue	28	23(82.1)	5(17.9)	

*P* <0.05 was considered statistically significant

**Table 3:** Surface Plasmon Resonance Method shows the dissociation constant of PRLR-DbsAb and its control mAb against recombinant target antigen

Antibody(Antigen)	Ka(1/Ms)	Kd(1/S)	KD(M)
PRLR mAb(PRLR)	3.98E+5	1.62E-4	4.07E-10
PRLR-DbsAb(PRLR)	2.94E+5	6.79E-4	2.31E-9
CD3 mAb(CD3)	9.92E+5	8.96E-4	9.03E-10
PRLR-DbsAb(CD3)	2.47E+5	2.06E-2	8.36E-8

### PRLR-DbsAb activates T cells resulting in Cytokine release

**Fig. 4:**
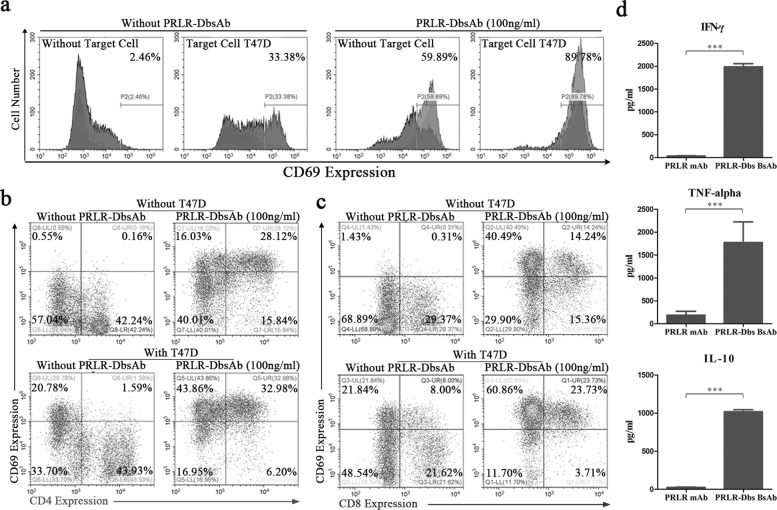
PRLR-DbsAb activates T cells resulting in Cytokine release. Effector cells (huPBMCs) and target cells (T47D) were incubated with 100 ng/ml recombinant antibodies (PRLR mAb and PRLR-DbsAb) for 20 h (effect-to-target ratio of 10:1). The cells were stained by FACS antibody to analyze the expression of activation marker protein CD69 on the surface of PBMCs (**a**), CD4 (**b**) and CD8 (**c**) T cells. And the secreted levels of IFN- *γ* (**d**), TNF- *α* (**e**) and IL10 (**f**) in the culture supernatant were detected by Elisa method. The experiment was repeated three times and the data was expressed as the Mean ±SEM, ***P <0.001

We next tested the activation of T cells by PRLR-DbsAb in vitro. When PRLR-DbsAb was incubated with target cells, the T cell activation marker CD69 in PBMCs **was upregulated** with 89.79% being positive while only 59.89% cells were positive for CD69 with PRLR-DbsAb alone without target cells (Fig. 4a). The activation of CD4+ and CD8+ T cells mediated by PRLR-DbsAb was further determined. The CD4+CD69+ T cells in PBMCs increased from 0.16% to 1.59% and CD8+CD69+ T cells increased from 0.31% to 8.00% with target cells only, showing target cells had more influence on CD8+ T cell activation (Fig. 4, b and c). Interestingly, The CD4+CD69+ cells increased from 0.16% to 28.12% and CD8+ CD69+ positive cells from 0.31% to 14.24% with PRLR-DbsAb only (Fig. 4, b and c), indicating PRLR-DbsAb could activate both CD4+ and CD8+ T cells independently. At the presence of both target cells and PRLR-DbsAb, the percentage of CD4+CD69+ and CD8+CD69+ cells reached 32.98% and 23.72% (Fig. 4, b and c). These results demonstrated CD4+ T cell activation was mainly dependent on PRLR-DbsAb while CD8+ T cell activation was relied on the combined action of target cells and PRLR-DbsAb. These results supported that CD8+ T cells are the main effector cells during lymphocyte-mediated killing of tumors [33]. It is well documented that inflammatory cytokine release is necessary for an effective tumor immunotherapy, especially those associated with Th1 cell polarization [34]. To assess whether favorable cytokines were produced in the process of T cell activation by PRLR-DbsAb, we measured the IFN- *γ*, TNF- *α* and IL10 levels in the cell culture supernatant when effector cells were incubated with target cell and PRLR-DbsAb or PRLR mAb. The cytokine release was barely detectable with PRLR mAb while the levels were dramatically increased with PRLR-DbsAb (Fig. 4d-f). In conclusion, PRLR-DbsAb activates T cell resulting in cytokine release.

**Fig. 5:**
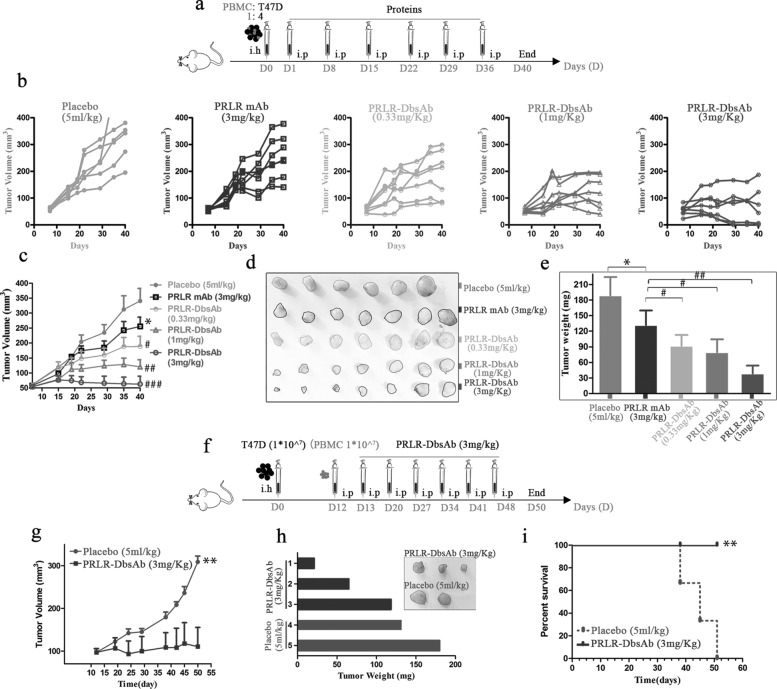
PRLR-DbsAb inhibits PRLR-expression tumor growth in vivo. sc Tumor cells plus sc effector cells (E/T 1:4) model: (**a**) Schematic schedule of inoculating tumor and treatment. A total of 1* 10^7^ T47D cells and 2.5* 10^6^ unstimulated huPBMCs were injected into mice and inoculated mice were administered with weekly intraperitoneal administration of 5 ml/kg PBS, 3 mg/kg PRLR, 0.33 mg/kg, 1 mg/kg, or 3 mg/kg PRLR-DbsAb (n=6-7); (**b**) T47D tumor sizes. Data were presented as measured tumor volume from different mouse; (**c**) T47D tumor sizes. Data were presented as Mean ±SEM ; (**d**) The digital image of stripped tumor; (**e**) Stripping tumor weight. Sc tumor cells plus ip effector cells (1:1) model: (**f**) Schematic schedule of inoculating tumor and treatment; (**g**) T47D tumor sizes. Data were presented as Mean ±SEM (n=3); (**h**) the digital image and weight of stripped tumor; (**i**) Mouse growth curve. * and ** mean compared with the placebo group, *P <0.05 and **P <0.01. ^*#*^, ^*##*^ and ^*###*^ mean compared with the PRLR mAb group, ^*#*^*P* <0.05, ^*##*^*P* <0.01 and ^*###*^*P* <0.001.

### PRLR-DbsAb inhibits tumor growth in vivo in immunocompromised mice

The in vivo activity of PRLR-DbsAb was studied in NOD/SCID mice using preventive or therapeutic administration. First the T47D cells expressing PRLR together with huPBMCs from a healthy donor were subcutaneously implanted into immunodeficient mice. PRLR mAb or PRLR-DbsAb was intraperitoneally administrated once per week starting the second day after tumor implantation (Fig. 5a). Compared with placebo group, PRLR mAb at a dosage of 3 mg/kg exhibited a slight inhibition of tumor growth, which was comparable to PRLR-DbsAb with a low dosage of 0.33 mg/kg. Moreover, PRLR-DbsAb could significantly inhibit the volume and weight of T47D derived-tumor and the effect was dose dependent (Fig. 5, b-e). Next we inoculated T47D cells into the oxter in mice. After 12 days, 1* 10^7^ inactivated huPBMCs were intraperitoneally injected and 3 mg/kg PRLR-DbsAb the following day (Fig. 5f). Mice with PRLR-DbsAb showed a better tumor inhibition and survival (Fig. 5g-i). These data illustrated that systematic administration of PRLR-DbsAb could inhibit the PRLR-expressing T47D xenograft tumor growth and increase survival time in mice.

### PRLR-DbsAb stimulates T cell infiltration and the PD-L1 expression in tumor tissue

**Fig. 6:**
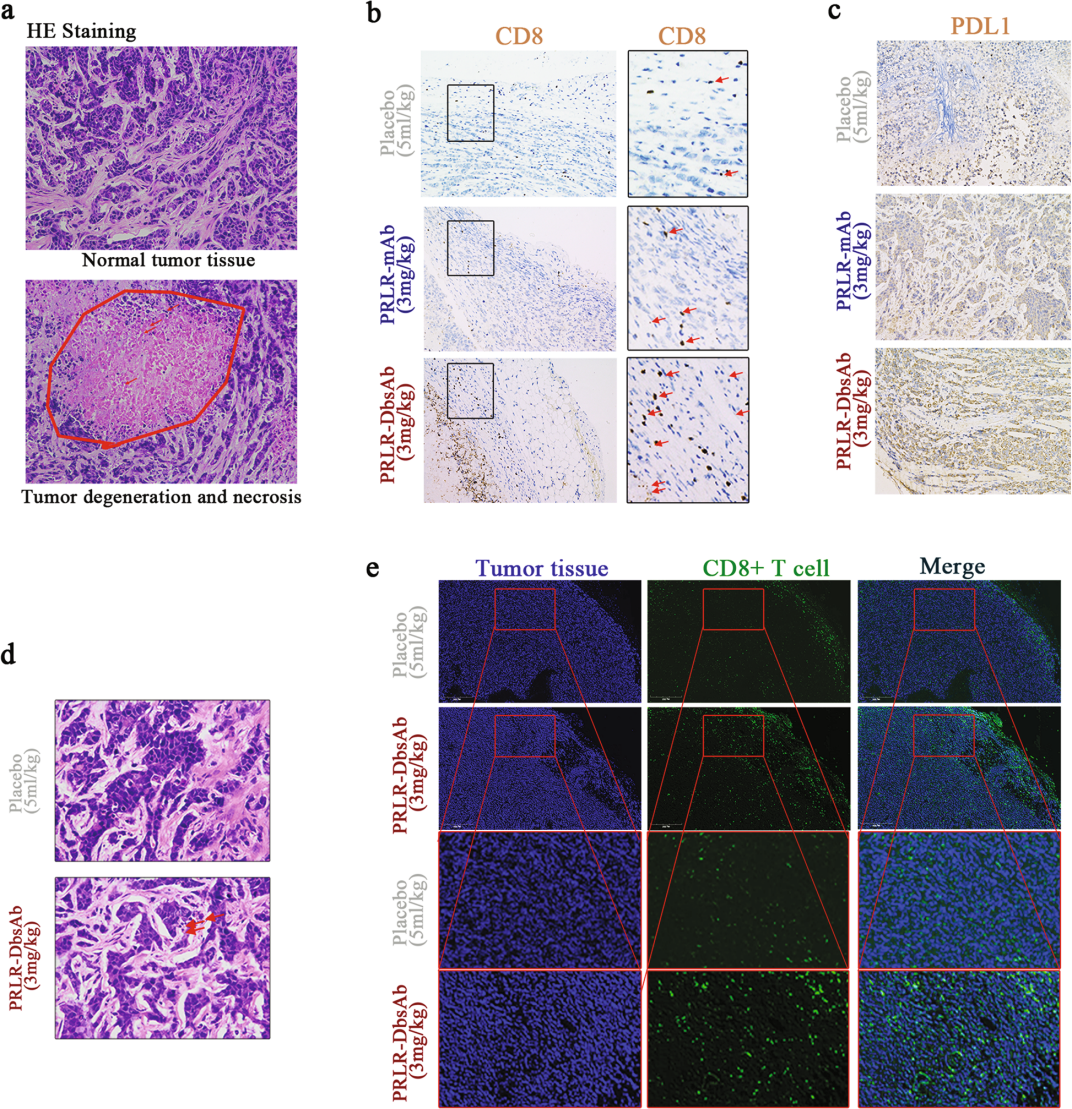
PRLR-DbsAb stimulates T cell infiltration and the PD-L1 expression unregulated in tumor tissue. sc Tumor cells plus sc effector cells (E/T 1:4) model: (**a**) HE staining of tumor tissue strapped from mice treated with 3 mg/kg PRLR-DbsAb. The above image showed normal tumor tissue, and the following image showed degenerate necrotic tumor tissue; (**b**) Immunohistochemical staining of CD8 in tumor tissue from the mice of different group; (**c**) Immunohistochemical staining of PD-L1 in tumor tissue from the mice of different group. Sc tumor cells plus ip effector cells (1:1) model: (**d**) HE staining; (**e**) Immunofluorescent staining of CD8. Three independent experiments were conducted and representative data is shown in this figure

Immune cells were found throughout the denatured and necrotic tumor tissue in a 3 mg/kg PRLR-DbsAb treated mice (Fig. 6a). The tumor immunofluorescence showed slight infiltration of CD8+ T cells of tumor tissues in mice treated with PBS, and they were gathered around the blood vessels of tumor tissues (Fig. 6e). We found little CD8+ T cells survived in tumor tissues of PBS-treated mice that was co-injected with PBMCs and tumor cells (Fig. 6b). In contrast, immunofluorescence of tumor tissues in PRLR-DbsAb treated mice showed more CD8+ T lymphocytes distributing dispersedly around the tumors (Fig. 6e) and more surviving T cells in tumor tissues (Fig. 6b). PD-L1 was reported to be involved with one of main mechanisms for immune escape for breast cancer [35*,*^36^]. We also analyzed the PD-L1 expression in tumor tissues with immunohistochemistry. Compared to Placebo and PRLR mAb group, PRLR-DbsAb treated mice exhibited obvious PD-L1 expression (Fig. 6c). In conclusion, PRLR-DbsAb could stimulate the infiltration of effector T cells as well as the expression of PD-L1 for tumor cells.

### PD-1 inhibition increases PRLR-DbsAb mediated cytotoxicity of PD-L1 positive target cell

**Fig. 7:**
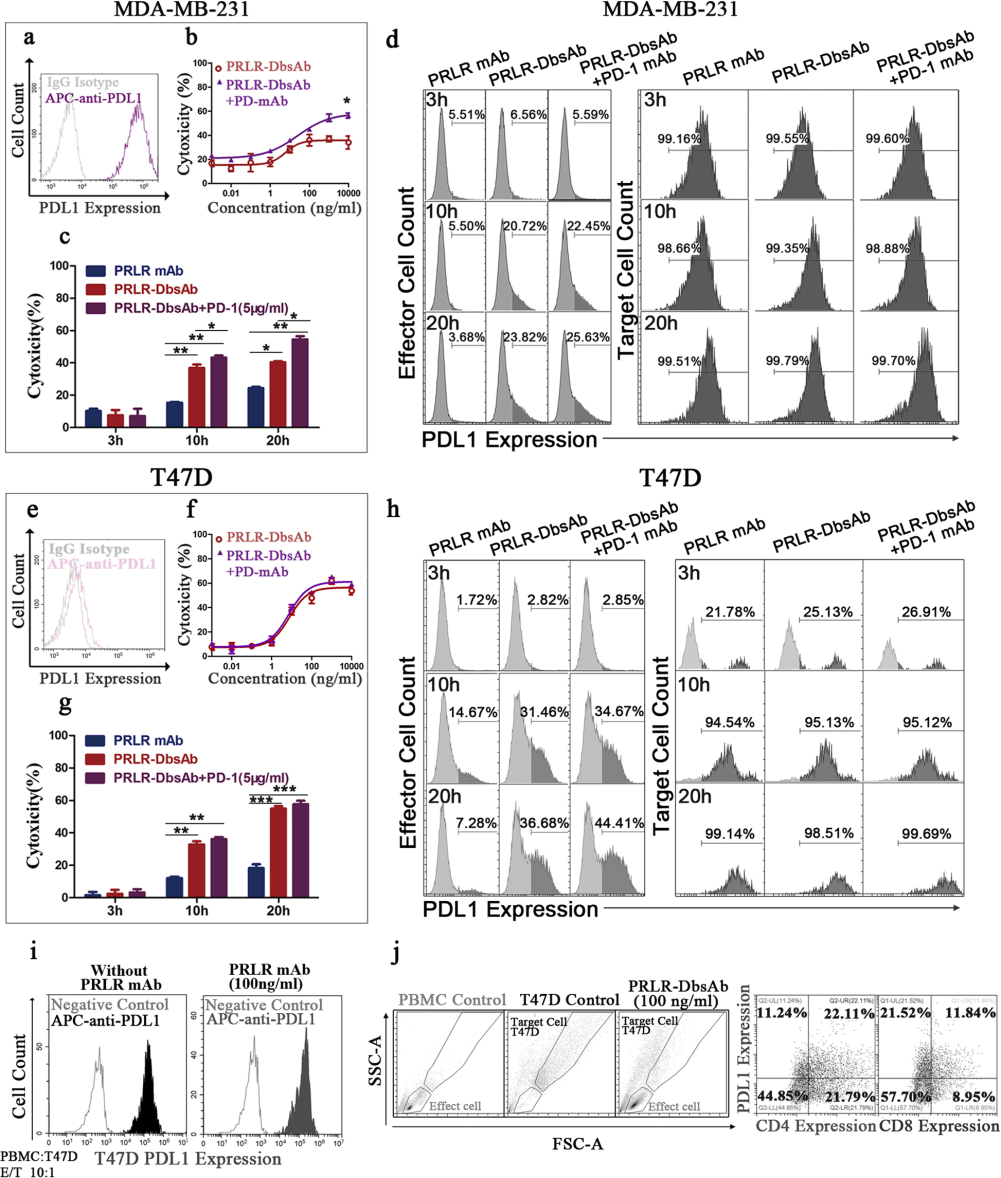
PD-1 inhibition increases PRLR-DbsAb mediated cytotoxicity of PRLR expression target cell. FACS histograms of PDL1 expression on different breast cancer cell lines including MDA-MB-231 (**a**) and T47D (**e**). The gray histogram is the fluorescent signal of non-specific IgG control antibody. Freshly isolated PBMCs and the target cells of MDA-MB-231 (**b**) or T47D (**f**) were incubated with or without addition of PD-1 mAb in different concentrations of PRLR-DbsAb at the ratio of 10:1 (E: T) for 20 hours. MDA-MB-231 (**c**) and T47D (**g**) cells were treated with 100 ng/ml PRLR-DbsAb at different time points (3, 10 and 20h) with or without adding PD-1 mAb and LDH release was detected. The expression of PD-L1 (**d** and **h**) on the corresponding effector cells and target cells is measured by Flow cytometer, respectively. (**i**) FACS histogram of the PD-L1 expression of target T47D with or without addition of 100 ng/ml PRLR mAb. (**j**) PD-L1 expression on CD4+ and CD8+ T cells. PBMCs and target T47D cells were incubated with 100 ng/ml PRLR-DbsAb at the ratio of 10:1 (E: T) for 20 hours. Three independent experiments were performed and the data was represented as the Mean ±SEM. *, ** and *** respectively indicate a statistically significant difference of at least *P* <0.05, <0.01 and <0.001

MDA-MB-231 is a model breast cancer cell line with high expression of PD-L1 (Fig. 7a). Therefore it could serve as a cell line in which cytotoxicity of bispecific antibody could be assessed with a blockade of PD-1. PD-1 mAb alone could enhance the cytotoxicity of effector cells against MDA-MB-231 (Fig. 7b), indicating the selected PD-1 mAb had a good bioactivity. The best killing activity of PRLR-DbsAb against MDA-MA-231 was 35% while it was 21% for PRLR mAb (Fig. 2h), showing PRLR-DbsAb could mediate the cytotoxicity of cells compared to PRLR mAb. Combined with PD-1 mAb, the best killing activity reached to 55% (Fig. 7b and c), which demonstrated blockade of PD-1 might enhance the PRLR-DbsAb-mediated cytotoxicity of against tumor cells with high level of PD-L1. In T47D xenograft mice models, the PDL1 expression was upregulated. We measured the cytotoxicity under different conditions after 20 h incubation of PBMCs with T47D cells. Compared to PRLR-DbsAb alone, the combination with PD-1 mAb group showed an increasing trend of cytotoxicity without significant difference (Fig. 7f). We further analyzed the cytotoxicity and the expression of PD-L1 on effector cells and target cells after incubation for 3 h, 10 h and 20 h. It was found PD-1 mAb could enhance the PRLR-DbsAb mediated cytotoxicity of against MDA-MB-231 after incubation for 10 h (*P <0.05*), and this enhancement was further pronounced after 20 h incubation, but not for T47D cells (Fig. 7, d and f). Interestingly, PD-L1 expression on effector immune cells was found to increase gradually with the incubation being longer as the MDA-MB-231 and T47D cells were treated with PRLR-DbsAb (Fig. 7d and h). This was not found in the PRLR-mAb treated group, indicating PRLR-DbsAb could stimulate the immune cells to express PD-L1. More experiments showed that the PD-L1-expression effector cells were CD4+ and CD8+ T cells (Fig. 7j). Moreover, the PD-L1 was remarkably expressed on T47D cells but not MDA-MB-231 cells during the incubation (Fig. 7d and h), corresponding to the result of PD-L1 expression in tumor tissue with IHC (Fig. 6). All above results showed that inhibition of PD-1 could enhance the cytotoxicity of PRLR-DbsAb against breast cells with high level of PD-L1.

## Discussion

Breast cancer is still a big threat to women’s health accounting for 30% of newly diagnosed cancers in great medical demand [37]. It’s confirmed that prolactin receptor plays an important role in the development of breast cancer [38]. Immunotherapy for the treatment of cancer get more attention recently. The BsAbs which could redirect the T cells to tumor cells and kill them showed to be a highly promising strategy both in pre-clinical and clinical sets [11*–*14*,*^19^*–*21]. And here in our study, we generated a bispecific antibody targeting the PRLR and CD3 exhibiting a favorable activity both in vitro and in vivo. Agarwal *et al* has reported a negative result of PRLR mAb LFA102 (a kind of humanized mAb binding with PRLR to inhibit the PRLR-mediated signaling transduction) for the treatment of metastatic PRLR positive breast cancer and metastatic castration resistant prostate cancer in phase I clinical trials (10). In our study, PRLR-DbsAb could enhance the cytotoxicity by more than 2 times relative to the top cytotoxicity observed for PRLR mAb (Fig. 2h). The dosage of PRLR-DbsAb needed for achieving 30% killing of the PRLR highly expressing cell lines is only 10 ng/ml (pM) while it is 1000 ng/ml for PRLR mAb, which is 100 times higher (Fig. 2f). Tumor-bearing mice were used to investigate the antitumor activity of PRLR-DbsAb treatment with intraperitoneal (i.p) injection because there is no differences in the peak plasma concentrations between i.p and i.v administration of antibody in mice [39] and i.p administration is relatively easy to perform the in vivo experiment. In vivo results illustrated that 0.33 mg/kg PRLR-DbsAb had the comparable anti-tumor activity with PRLR mAb at a dosage of 3 mg/kg (Fig. 7c). The Fig. 2h experiment had shown from in vitro perspective that the anti-tumor effects of PRLR-DbsAb were not superimposed by the anti-tumor effects of CD3 and PRLR monoclonal antibody. The in vivo experiment would be more helpful to illustrate the effectiveness of PRLR-DbsAb using the mixture of both monoclonal antibodies as the control group, which will be reported in our future in-depth study. We used the NOD/SCID mouse model to assess the PRLR-DbsAb activity in vivo due to its unique advantage in evaluating candidate drug in human tumor transplanted animal systems. However, there is a main disadvantage of this model, that it is not able to provide human immune cells which usually have very limited half-life in the murine background [40]. In addition, we injected huPBMCs with peritoneal administration, which could further exacerbate this problem. Taking these factors into consideration, we believe the full potential of PRLR-DbsAb is underestimated, especially at the thought of a great number of T cells circulating in the human body in clinical sets. PRLR is overexpressed in certain types of breast cancer as well as prostate cancer. Unlike another target for breast cancer HER2 (expressed in most normal epithelial cells), PRLR is only slightly expressed in normal breast tissues. In the treatment of advanced breast cancer, surgery is routinely performed to remove the potentially infiltrating breast tissue [41,42]. In Phase I clinical climbing trials (3-60 mg/kg), 73 patients were treated, including 53 at the highest dose level. Overall, LFA102 was safe and well-tolerated; the maximum tolerated dose was not reached, as there were no dose-limiting toxicities [6]. These researches indicated minor severe side effects of PRLR immunotherapy. PD-L1 which is expressed on many cancer and immune cells, plays an important role in blocking the ‘cancer immunity cycle’ by binding to PD-1 and B7.1 (CD80), both negatively regulate T-lymphocyte activation. Binding of PD-L1 to its receptors suppresses T-cell migration, proliferation and secretion of cytotoxic mediators, and restricts tumor cell killing [43,44]. Triple negative breast cancer (TNBC) expressing PD-L1 participates in tumor immune escape by binding PD-1 to inhibit T cell activation [45,46]. Here, our in vitro experiments showed that inhibition of PD-1 enhanced PRLR-DbsAb killing of PRLR low expression TNBC cells. PD-L1 weakly expressed on cell line gradually unregulated with the prolongation of cell killing time mediated by T cell, suggesting that PD-L1 expression level in response to immunotherapy. PD-L1 expression is predictive of a clinical response to anti-PD-1/PD-L1 therapies [47]. And there are extensive CD8+/PD-L1+ immune infiltration in breast cancer [48]. It is noteworthy that a response to PD-1/PD-L1 therapy is strongly associated with immune cell with PD-L1 expression rather than tumor cell with PDL1 expression [49]. Even more surprisingly in the study, PRLR-DbsAb significantly up-regulated PDL1 expression on CD4+ and CD8+ T cells compared to PRLR mAb treatment. The results of ex vivo experiments showed that the immunotherapeutic bispecific antibody PRLR-DbsAb may suppress the immune response by upregulating PD-L1 in lymphocyte besides PD-L1 in tumor. More in vivo and in vitro studies need to be conducted to investigate the role of PD-1/PD-L1 signaling pathways in the treatment of tumors with bispecific antibodies.

## Conclusions

Our studies demonstrated that the PRLR-DBsAb redirected T cells to breast tumor cells and showed better anti-tumor activity rather than PRLR antibody. This finding could provide a novel strategy for the treatment of metastatic breast cancer and prostate cancer based on PRLR target. Our results also suggest the benefits of a combination of two immunotherapies: recruiting T-cell and inhibiting of T-cell inhibitory PD-1/PD-L1 signaling, may enhance the immune response.

## Supplementary information

**Additional file 1Figure S1**. Recombinant protein preparation. (a) SDS-PAGE analysis of Protein L affinity chromatography purified antibody fragment B under reduced and non-reduced conditions with commassive staining. SDS-PAGE analysis of the reaction mixture of fragment A and B in the presence of 0.5 mM DTT for 4 h at 4 ^∘^C. under reduced (c) and non-reduced (b) conditions. (d) SDS-PAGE analysis of the Protein A elutions under reducing and non-reducing conditions. 8 to 14, Protein A elutions. Please note that the reaction mixture was diluted 20 times and then analyzed by SDS-PAGE gel, so the band is very light and may appeared unclear.

**Additional file 2Figure S2**. PRLR protein is expressed on breast cancer. (a) PRLR protein expressions in tissue chip (30 paired breast cancer and para-carcinoma tissues) were measured with immunohistochemistry. (b) Qualitative expression of PRLR protein. (c) Three representative examples of PRLR expression on breast cancer and para-carcinoma tissues (×200).

## Data Availability

All data generated or analysed during this study are included in this published article.

## Abbreviations

ADC: Antibody-drug conjugate
BAPTS: Intein mediated protein trans-splicing system
BiTE: Bispecific T-cell engager
CAR-T: Chimeric antigen receptor T-cell
FBS: Fetal bovine serum
mAb: Monoclonal antibody
PBMC: Human peripheral blood mononuclear cell
PBS: Phosphate buffer saline
PRLR: Prolactin Receptor
SDS-PAG: Sodium dodecyl sulfate-polyacrylamide gel electrophoresis
TAA: Tumor associated antige
TGE: Transient gene expression
TNBC: Triple negative breast cancer
