# Supplementary figures and images for: A novel bispecific antibody targeting CD3 and prolactin receptor (PRLR) against PRLR-expression breast cancer
Source: J Exp Clin Cancer Res. 2020 May 12;39:87. doi: 10.1186/s13046-020-01564-4 (PMC7216678; doi:10.1186/s13046-020-01564-4)

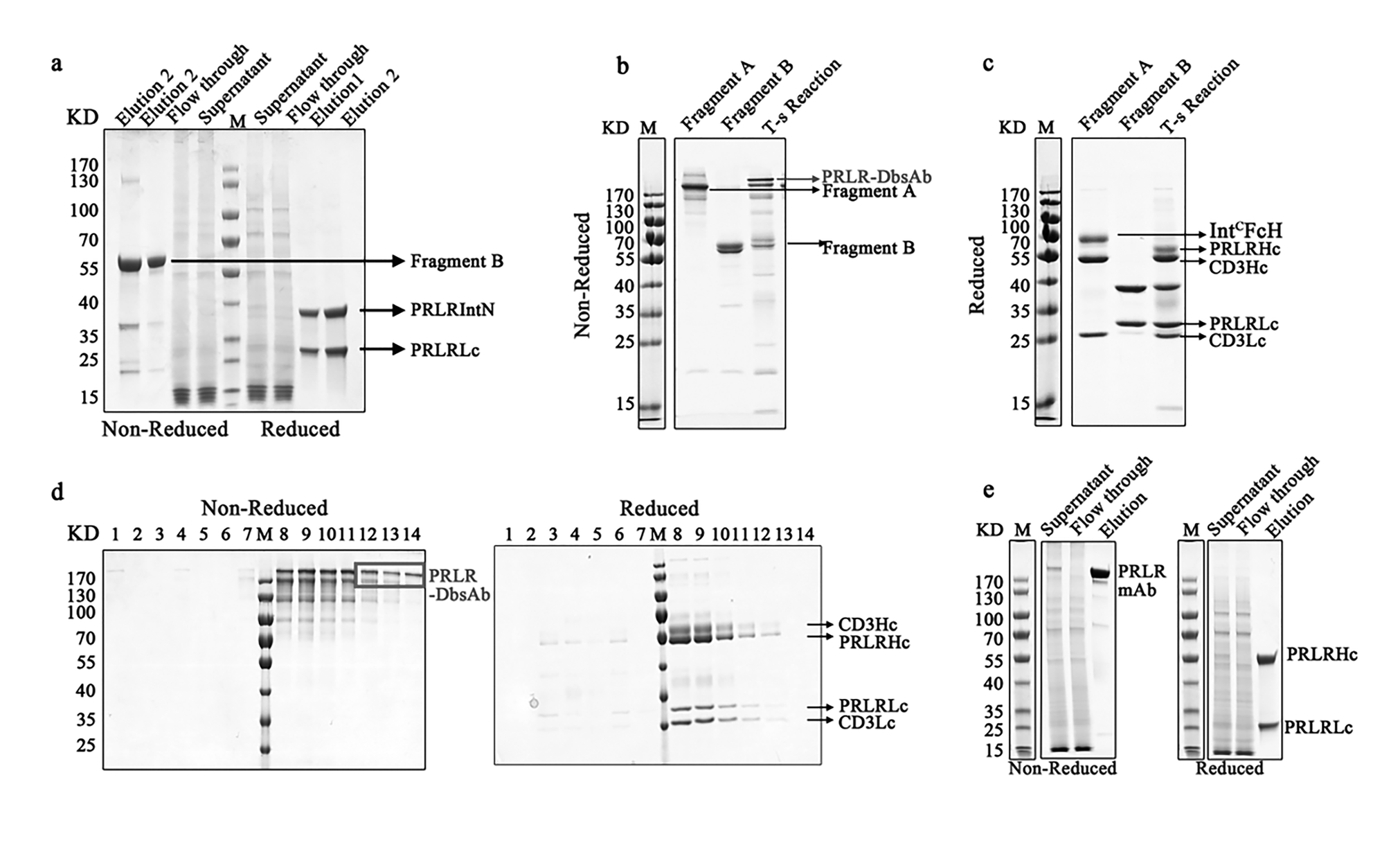

Supplement: Supplementary file 1 — Additional file 1Figure S1. Recombinant protein preparation. (a) SDS-PAGE analysis of Protein L affinity chromatography purified antibody fragment B under reduced and non-reduced conditions with commassive staining. SDS-PAGE analysis of the reaction mixture of fragment A and B in the presence of 0.5 mM DTT for 4 h at 4 ∘C. under reduced (c) and non-reduced (b) conditions. (d) SDS-PAGE analysis of the Protein A elutions under reducing and non-reducing conditions. 8 to 14, Protein A elutions. Please note that the reaction mixture was diluted 20 times and then analyzed by SDS-PAGE gel, so the band is very light and may appeared unclear. [file 13046_2020_1564_MOESM1_ESM.tif]

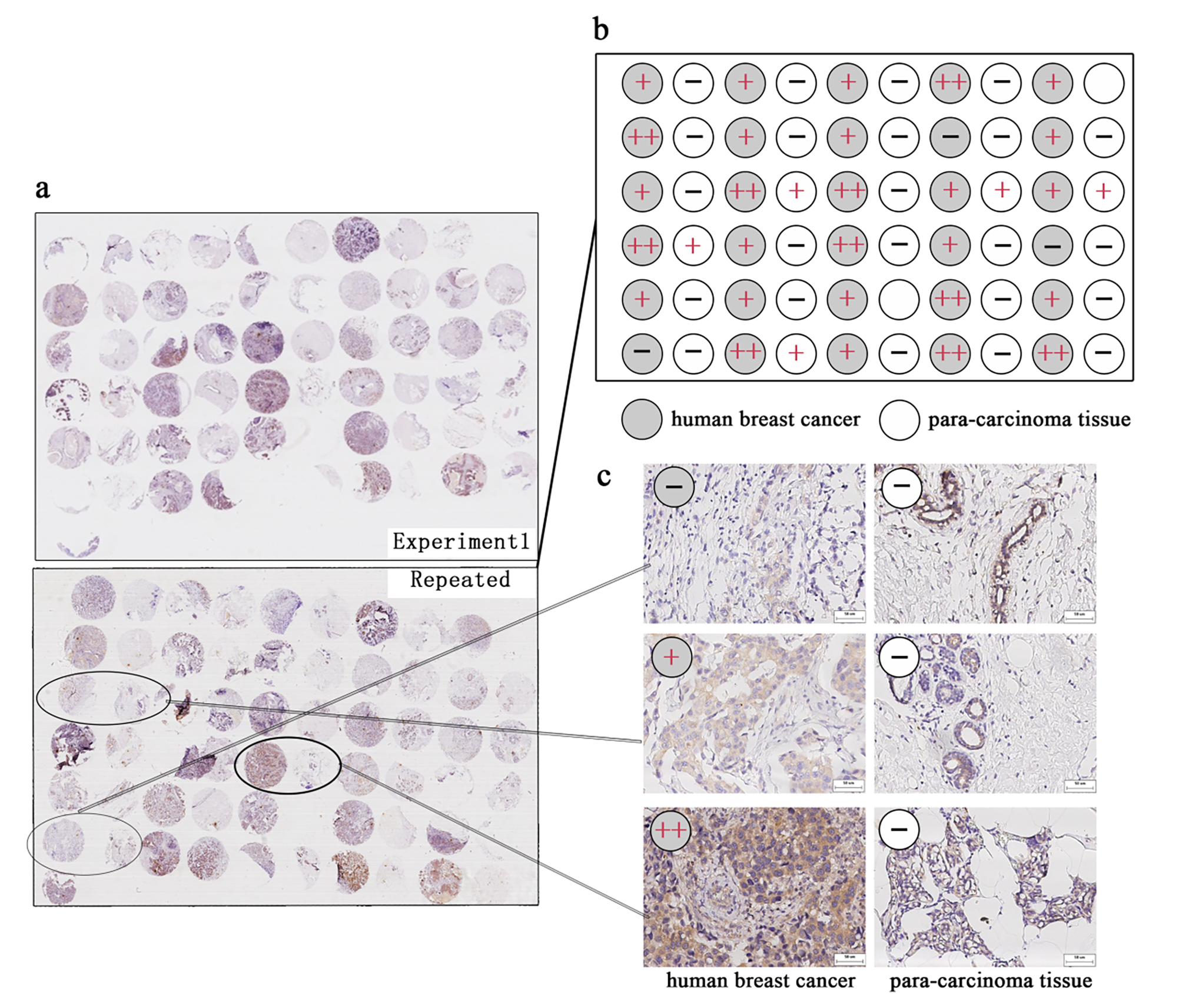

Supplement: Supplementary file 2 — Additional file 2Figure S2. PRLR protein is expressed on breast cancer. (a) PRLR protein expressions in tissue chip (30 paired breast cancer and para-carcinoma tissues) were measured with immunohistochemistry. (b) Qualitative expression of PRLR protein. (c) Three representative examples of PRLR expression on breast cancer and para-carcinoma tissues (×200). [file 13046_2020_1564_MOESM2_ESM.tif]
